# Properties of Doped GaSb Whiskers at Low Temperatures

**DOI:** 10.1186/s11671-017-1923-1

**Published:** 2017-02-27

**Authors:** Igor Khytruk, Anatoly Druzhinin, Igor Ostrovskii, Yuriy Khoverko, Natalia Liakh-Kaguy, Krzysztof Rogacki

**Affiliations:** 1 Lviv Polytechnic National University, 12 S. Bandera Str., 79013 Lviv, Ukraine; 2 International Laboratory of High Magnetic Fields and Low Temperatures, Gajowicka 95, Wroclaw, Poland

**Keywords:** Magnetoconductance, n-type GaSb whiskers, Weak antilocalization, Superconductivity

## Abstract

Temperature dependencies of GaSb whiskers’ resistance doped with Te to concentration of 1.7 × 10^18^ cm^−3^ were measured in temperature range 1.5–300 K. At 4.2 K temperature, a sharp drop in the whisker resistance was found. The observed effect is likely connected with the contribution of two processes such as the electron localization in the whiskers and transition in superconducting state at temperature below 4.2 K. The whisker magnetoconductance is considered in the framework of weak antilocalization (WAL) model and connected with subsurface layers of the whiskers. The Shubnikov-de Haas (SdH) oscillatory effect is observed in high-quality n-type GaSb whiskers with tellurium doping concentration near the metal-insulator transition (MIT) for both longitudinal and transverse magnetoresistance.

## Background

GaSb is an important material for practical implementation in microelectronic circuits, sensors, solid-state lasers, electrooptic modulators, and other new devices due to the high quality and perfection of single crystals [1, 2]. The GaSb epitaxial layers, or quantum dots grown on lattice-matched, semi-insulating substrates, are used in high-speed devices due to their advantages such as low density of dislocations and defects [3, 4]. However, these structures are affected by the substrate which restricts the advantages. Hence, GaSb single crystals [5] and free-standing nano- and microwires [6] were used to avoid the abovementioned shortcoming. GaSb nanowires can also be used for lasing devices [7] that open the possibility of their applications for semiconductor subwavelength-wire lasers in photonic integrated circuits. The high charge carrier mobility and dimension dependence of conductance in GaSb∕GaAs nanowire heterostructures allow their wide usage [8, 9]. The high-performance GaSb nanowires could be integrated with devices on the base of InSb, InAs, and InGaAs nanowires by the various transfer techniques [8, 10].

Whiskers have several advantages over the wires, in particular, such as absence of defects due to the single-crystal structure of whiskers caused by their growth conditions. Despite their large dimensions (as compared with nanowires), whiskers are important objects of nanoscale studies taking into account their core-shell structures [11]. New physical effects were obtained when the whiskers’ doping concentration corresponded to metal-insulator transition, for instance, giant piezoresistance effect in Si whiskers [12], appearance of magnetophonon oscillations in Ge whiskers [13], and giant spin-orbit interaction with g-factor 60 in InSb whiskers [14]. However, GaSb whiskers were rarely studied from this point of view.

The aim of this work is to study spin-orbit interaction for understanding observed superconductivity and weak antilocalization in n-type GaSb whiskers with Te impurity concentration near the metal-insulator transition by measurement of the magnetoresistance and magnetoconductance in the temperature range 1.5–300 K and magnetic field 0–14 T.

## Methods

n-type GaSb whiskers doped with Te were selected for the study. The whiskers were grown by chemical vapor deposition (CVD) method in a closed system [15]. The whisker’s diameter ranges from 15 to 20 μm with length from 4 to 5 mm. Current electrical contacts were created by welding Pt whiskers on the opposite ends of the studied whiskers. The distance between potentiometric contacts was equidistant and equal to 1 mm. The method provides resistive contact to the samples in the temperature range 4.2–300 K. As a result, linear *I*-*V* curves were obtained. Te is commonly used as n-type dopant in GaSb for creating the photovoltaic devices. The results of doped whiskers investigation by ion mass spectroscopy have shown that n-GaSb whiskers (ρ_300K_ = 0.004 Ω cm) have Te concentration corresponding to the metallic side of metal-insulator transition and it is equal to 1.7 × 10^18^ cm^−3^. To obtain reproducible results, a group of approximately ten whiskers with identical characteristics was studied. The samples have been cooled down to 1.5 K in helium cryostat. A special inset with a bifilar winding heater has been used to heat-up samples to room temperature. Stabilized electrical current of 1–100 μA depending on the resistance of the sample has been generated by Keithley 224 current source. Digital multimeters Keithley 2000 and Keithley 2010, with simultaneous automatic data registration via parallel port of PC, have been used to measure both the voltage at potential contacts of the samples, output signals from thermocouple and magnetic field sensor with accuracy up to 1 × 10^−6^ V. The Bitter magnet-based setup has been used to study the effect of strong magnetic field on the samples. The induction of the magnet increases from *B* = 0 up to *B* = 14 T at a rate ~1.75 and 3.5 T/min at 4.2 K and higher temperature range, respectively.

## Results

**Fig. 1:**
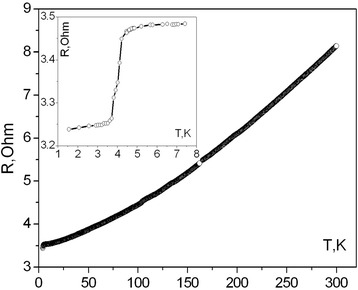
Temperature dependence of GaSb whisker resistance in the temperature range 1.5−300 K in a zero magnetic field. *Inset*: temperature dependence of GaSb whisker resistance for low temperatures down to 1.5 K

Temperature dependence of resistance for Te-doped GaSb whisker at a zero magnetic field is shown in Fig. 1 over the temperature range 1.5–300 K. Reviled *R* (*T*) with monotonic decrease of the resistance from 8 to 3.5 Ω in the temperature range 4.2–300 K corresponds to the metallic behavior of the electric resistivity of the GaSb whiskers. Unexpectedly the sudden decrease of GaSb whisker resistance from 3.5 to 3.25 Ω is observed at temperature just below 4.2 K. This interesting behavior of the resistance, namely, the sudden drop of *R* (*T*) at *T* ~4.2 K, could indicate the superconducting transition at this low temperature (inset in Fig. 1). To be sure, firstly, it should be discussed to what extent the junction properties (welded Pt microwires) influence the measurement data at about 4 K and may contribute to the observed drop in the resistance effect. To check the possible influence of Pt junctions, *I*-*V* characteristics of the whiskers were measured in the temperature range 1.5–4.2 K. The measurements have shown that *I*-*V* characteristics were linear at every fixed temperature and electrical contacts from Pt microwires remain ohmic. Thus, a sudden drop in the whisker’s resistance is hardly connected with influence of the Pt contacts at low temperatures.

**Fig. 2:**
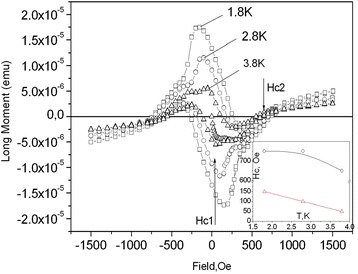
Magnetization of GaSb whiskers versus magnetic field induction at various fixed temperatures. *Inset*: temperature dependencies of *H*_c1_ (Δ) and *H*_c2_(○)

On the other hand, the drop in resistance is very small, namely, about 8.6% ((3.5–3.2)/3.5 = 8.6%), and the resistance below 4 K is still very large in order to be interpreted as transition to the superconducting states, which expectedly is associated with a zero resistance. Nevertheless, one can suppose a presence of a small superconductive phase in the whisker. To support the idea, one can examine a diamagnetic response of GaSb<Te> whiskers below 4.2 K in small magnetic fields. Magnetization of GaSb whiskers versus magnetic field with strength from −1.5 up to 1.5 kOe at various fixed temperatures was measured using SQUID technics and presented in Fig. 2. As can be seen from Fig. 2, the hysteresis, being typical for type II superconducting state of GaSb whiskers, is observed in the available temperature range from 1.8 up to 4.2 K. The data in Fig. 2 allows estimation of critical values of *H*_c1_ and *H*_c2_. All curves in Fig. 2 exhibit expected linear behavior at low fields (<250 Oe), which corresponds to the diamagnetic response of the sample. However, they deviate from the linearity at *H*_c1_, which is expectedly different for different temperatures. Temperature dependence of *H*_c1_ is shown in the inset of Fig. 2 (curve 2). On the other hand, we can also estimate *H*_c2_ at the point where magnetic field intensity corresponds to zero magnetization (see arrow in Fig. 2). Estimated values of *H*_c2_ as a function of temperatures are shown in the inset of Fig. 2. As can be seen from the graph, the maximal *H*_c2_ value is approximately 750 Oe.

Thus, the partial superconducting transition of GaSb whisker is likely to occur at the critical temperature of about 4.2 K. Nevertheless, the reason of the superconductivity in the whiskers is not clear, since both the *T*_c_ = 1.09 K of Ga as well as the Curie temperature for GaSb<Te> is substantially lower. The possible reasons of the superconductivity in the whiskers could be their amorphization [16] or the heavy Te doping up to concentrations in the vicinity to metal-insulator transition, which is known to increase substantially the *T*_c_ values [17]. The amorphization of the GaSb whiskers is excluded as was observed in Shubnikov-de Haas (SdH) oscillations, which are usually occurring in high-quality crystals. Metallic phase in the vicinity to the localization threshold of metal-insulator transition (MIT) could be responsible for anomalous superconducting transition in GaSb crystals at temperatures near 4 K [18]. The other possible reason of partial superconductivity could be the creation of Sb_2_Te_3_ nanoclusters, which are known to be topological insulators and to show a transition to superconducting state at *T*_c_ = 3 K [19]. However, Sb_2_Te_3_ nanoclusters are unlikely to be created in the whiskers taking into account the relatively low Te concentration (1.7 × 10^18^ cm^−3^ according to data of mass spectroscopy investigation). As a result, the number of formed potential Sb_2_Te_3_ nanoclusters is rather insufficient in order to provide the superconductive channel. Moreover, the partial superconductivity in the GaSb<Te> whiskers has been observed at *T*_c_ of about 4.2 K, which contradicts with *T*_c_ = 3 K observed for Sb_2_Te_3_ inclusions.

**Fig. 3:**
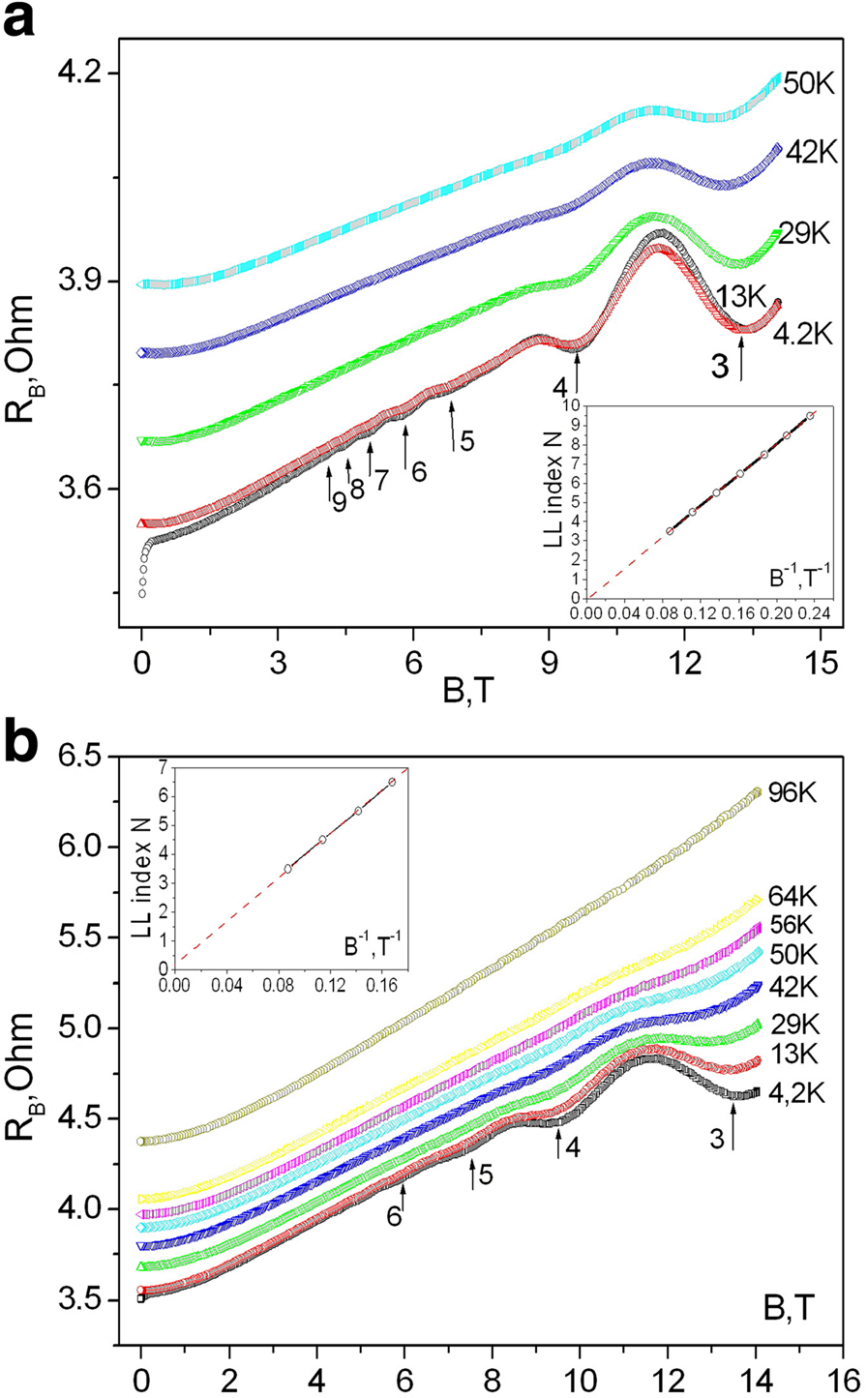
Longitudinal (**a**) and transversal (**b**) magnetoresistance of GaSb whiskers versus magnetic field induction at various fixed temperatures. The *arrows* correspond to numbered peaks of magnetoresistance oscillations. On the *insets*: the Landau level (LL) index N of SdH oscillation versus reverse magnetic field (1/B)

Suppression of superconductivity by magnetic field is also informative for explaining its possible origin. In order to observe the influence of magnetic field on the resistivity drop below 4.2 K, one has to investigate the GaSb<Te> whiskers magnetoresistance R_B_(*T*). Longitudinal and transverse magnetoresistance of n-type GaSb whiskers was studied in the temperature range 1.5–96 K and magnetic fields 0–14 T. Results of these investigations for GaSb whiskers with Te doping concentration 1.7 × 10^18^ cm^−3^, which correspond to metallic side of the metal-insulator transition, are presented in Fig. 3. The oscillation peaks are distinctly seen on both longitudinal (H || to the transport current *I*) (Fig. 3a) and transverse (H ⊥ to *I*) (Fig. 3b) magnetoresistance at low temperatures (Fig. 3). Corresponding values of magnetic field induction at *T* = 4.2 K for both longitudinal and transverse magnetoresistance oscillation peaks are marked by arrows in the figure. The maximum peak amplitude expectedly decreases with the increase of the temperature in the whole range of applied fields. However, it is still observed up to at least 50 K.

Seven oscillation peaks were revealed on longitudinal R_B_(B) dependence at liquid helium temperature (Fig. 3a). The number of transverse magnetoresistance peaks (Fig. 3b) decreases to four at 4.2 K in magnetic fields with induction up to 14 T. In our recent works, we found magnetophonon oscillations in n-Ge whiskers [13] and Shubnikov-de-Haas (SdH) oscillations in n-InSb whiskers in the temperature range 4.2–70 K [14]. In the present work, it is shown that the oscillations are observed in n-GaSb whiskers in the low temperature range. The oscillations are attenuated with the increase of temperature and are already absent at temperatures above 60 K. This fact indicates that the observed oscillations can be attributed to SdH oscillations like those in InSb whiskers [14].

Next, we follow the well-developed methodology and construct the Landau fan diagram [20]. To do this, we assign a Landau level (LL) index number N to each resistance minimum (N + 1/2 to each resistance maximum) as shown in Fig. 3a, b by arrows. The Landau level index N versus reversal magnetic field induction 1/B (Landau fan diagram) is shown in the insets of Fig. 3a, b. As it is obvious from the insets of Fig. 3a, b, the data points fall on a straight line and the solid line represents the best linear fit. The intercept of the linear fit with the N-index axis yields zero phase *β* = 0 that indicates in Schrodinger electron transport responsible for SdH oscillations.

## Discussion

### Magnetoresistance and Magnetoconductance of GaSb Whiskers in Low Magnetic Fields

**Fig. 4:**
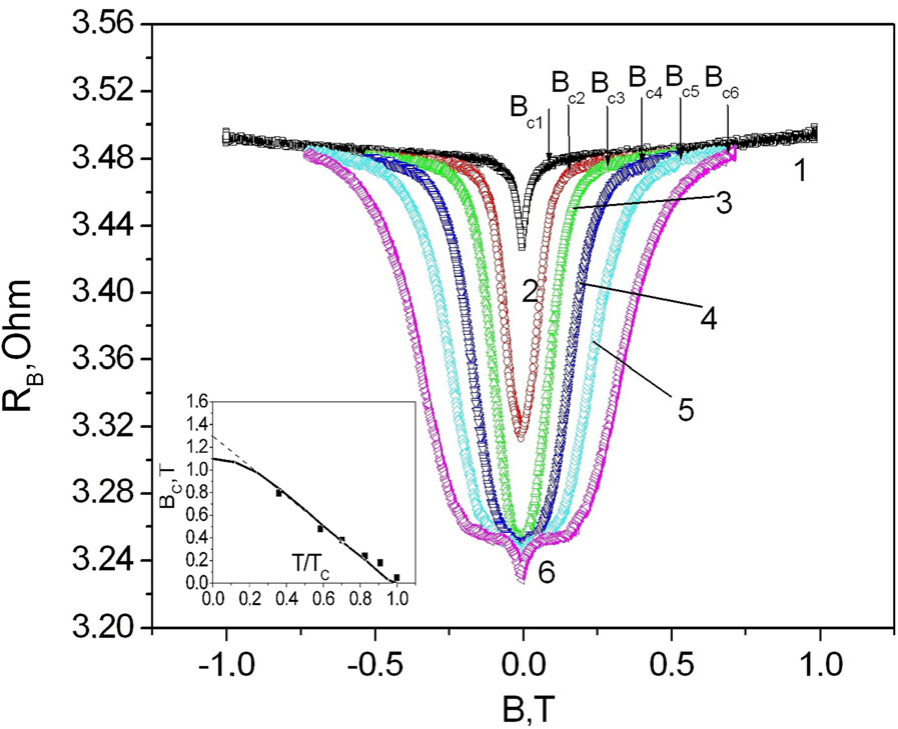
Magnetoresistance of GaSb whiskers at fixed temperatures: 1–4.2, 2–3.3, 3–3.47, 4–2.95, 5–2.45, and 6–1.5 K. The *arrows* designate the critical field *B*_c_ at fixed temperatures. *Inset*: the critical field *B*_c_ (*T*) versus reduced transition temperature *T*/*T*_c_ for GaSb whiskers. The *solid line* shows the Ginzburg-Landau fit to the experimental data

Let us consider the influence of low magnetic fields on GaSb<Te> whisker resistance in the temperature range 4.2–1.5 K (Fig. 4). As can be seen from the figure, the longitudinal magnetoresistance dependencies demonstrate the expressed cusps at fields up to 1.0 T. To gain a deeper understanding of the physics associated with the superconductivity, we extracted the upper critical field *B*_c_ (*T*) (denoted by arrows in Fig. 4) from the magnetoresistance curves obtained at fixed temperatures. The obtained *B*_c_ (*T*) versus *T*/*T*_c_ dependence is shown in the inset of Fig. 4. Meanwhile, the value of the upper critical field at absolute zero temperature *B*_c_ (0) was obtained by fitting the data to the generalized Ginzburg-Landau model:

1 $$ B\mathrm{c}(T)= B\mathrm{c}(0)\left(1-{t}^2\right)/\left(1+{t}^2\right), $$

shown by the solid curve in the graph, where *t* = *T*/*T*_c_.

Actually, the *B*_c_ (*T*) curve (inset of Fig. 4) exhibits a nearly linear dependence in the temperature range 1.5–4.2 K, as with topological YPtBi crystals [21]. Based on the linear dependence, the zero temperature upper critical field was obtained *B*_c_ (0) = 1.3 T by simple extrapolation (inset of Fig. 4, dash line).

The value of *B*_c_ (0) for GaSb is well below the Pauli paramagnetic limit:

2 $$ {B}_C^p(0)=1.85{T}_c\approx 7.7 K, $$

which suggests an orbital pair-breaking mechanism of superconductivity for GaSb whiskers [21].

Using the relation:

3 $$ {B}_{\mathrm{c}}(0)={\Phi}_0/2\pi \xi {(0)}^2, $$

where Φ_0_ is the flux quantum, we obtained superconductor coherence length ξ(0) = 1.7 nm. The obtained value is in good agreement with the coherence length *ξ*_ab_ (0) = 1.0 nm for cuprates [22].

The short coherence length (*ξ*(0) ~ 1.7 nm) of Cooper pairs, comparable with that found for high-*T*_c_ superconductors, could lead to a variety of fascinating phenomena in contrast to low-*T*_c_ materials, which have homogeneous superconducting properties on length scales *ξ*_0_ of the order of several hundreds of nanometers [22]. Thus, the obtained low value of the coherence length is believed to provide an evidence for a possible competition of superconductivity and other physical phenomena in GaSb whiskers.

A small diminution of the whisker resistance (8.6%) at *T* ≤ 4.2 K suggests the formation of only small superconductive islands in the sample, which causes the observed partial transition of the whisker to the superconducting state. Since the main part of the whisker conductance is believed to occur on the whisker surface, like that in Si whiskers [12], one can suppose that the partial superconductivity of the whiskers should take place on their surface. However, if the sample is biphasic, the superconductive transition is expected to be somewhat blurred. That is why the obtained narrow transition (sudden drop of the resistance at *T* ≤ 4.2 K) just might be an additional argument in favor of the surface superconductivity in the whiskers.

It is worthy to note that the value of *B*_c_ (0) for GaSb, obtained from the above measurements, is an order of magnitude greater than the value obtained from magnetization measurement (of about 0.075 T). Thus, the obtained magnetoresistance cusps cannot be satisfactory explained only by the whisker transition to the superconducting state. Another possible reason of the whisker magnetoresistance cusps could be the effect of weak antilocalization (WAL) similar to that observed in the single crystals based on Bi-Te [23].

The nature of WAL can be caused by spin-orbit interaction in the three-dimensional bulk channel. However, in GaSb whiskers, the two-dimensional nature of the electrical transports is assumed, as mentioned above, which is more likely to originate from the surface conductance, as it takes place in Si whiskers [12].

According to the WAL model [24], the electron-electron and electron-phonon scattering are supposed to emerge in the whiskers. The theoretical dependence of normalized magnetoconductance Δσ(*B*)/*G*_0_ for two-dimensional electron gas [24] has the following forms:

4 $$ \begin{array}{l}\frac{\Delta \sigma (B)}{G_0}=\frac{\sigma (B)-\sigma (0)}{G_0}= f\left(\frac{B}{H_{so}+{H}_{\phi}}\right)+\\ {}+\frac{1}{2} f\left(\frac{B}{2{H}_{so}+{H}_{\phi}}\right)-\frac{1}{2}\left(1+\beta \right) f\left(\frac{B}{H_{\phi}}\right),\end{array} $$

where *B* is the magnetic field induction and *β* is a factor determining the value of Maki-Thompson correction. Correspondingly,

5 $$ {G}_0={e}^2/2\pi h $$

is the quantum conductivity. Function *f*(*x*) is determined by the digamma function *Ψ*(*z*):

6 $$ f(z)=\Psi \left(\frac{1}{2}+\frac{1}{x}\right)+ \ln (x) $$

The parameter *H*_*ϕ*_ is related with the dephasing time *τ*_*ϕ*_ of the electron wave function caused by electron-electron or electron-phonon interaction:

7 $$ {H}_{\phi}=\frac{\mathit{\hslash c}}{4 eD{\tau}_{\phi}}. $$

Accordingly, the parameter *H*_*so*_ is related with the dephasing time *τ*_*so*_ caused by spin-orbit interaction of electrons:

8 $$ {H}_{so}=\frac{\mathit{\hslash c}}{4 eD{\tau}_{so}}, $$

where *c* is the light velocity and *D* is the diffusion coefficient.

**Fig. 5:**
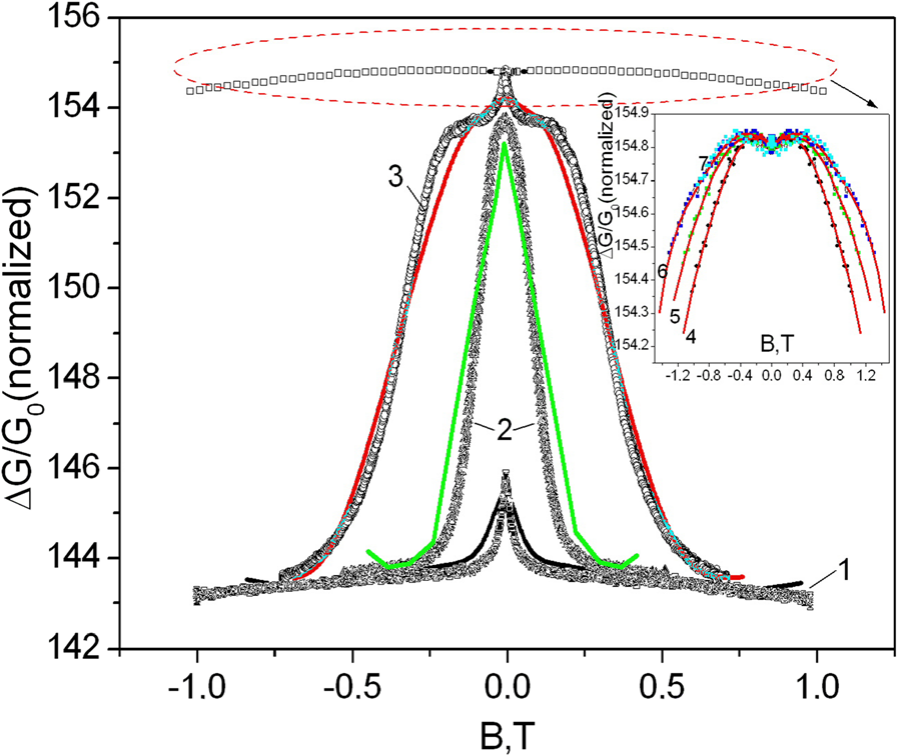
Magnetic field dependencies of normalized magnetoconductance change of GaSb whiskers at different temperatures (*dots*): 1–1.5, 2–3.3, 3–4.2, 4–12, 5–25, 6–42, and 7–50 K compared with theoretical curves calculated using (4) at the same temperatures

Further, let us consider *β* → 0. In this case, the conductivity change *Δσ*(*B*) in a magnetic field, normalized by the amount *G*_0_, was determined from experimental dependencies of GaSb whiskers’ magnetoresistance in magnetic field in the following way:

9 $$ \frac{\Delta \sigma (B)}{G_0}=\frac{\sigma (0)}{G_0}\left(\frac{\Delta R(B)}{R(0)}+{\left(\mu H\right)}^2\right), $$

where *μ* is the Hall mobility and *H* is the magnetic field intensity. The temperature dependencies of the Hall mobility for GaSb whiskers were calculated in Ref. [25]. Thus, the obtained dependence Δ*σ*(*B*) (Fig. 5) is matched with the theoretical one according to (4) in order to find the parameters *H*_*ϕ*_ and *H*_*so*_. Figure 5 shows the magnetic field dependencies of normalized magnetoconductance change Δσ(*B*)/*G*_0_ of GaSb whisker at different temperatures (dots): 1–1.5, 2–3.3, 3–4.2, 4–12, 5–25, 6–42, and 7–50 K compared with theoretical curves calculated using (4) at the same temperatures (solid lines). As it is seen from Fig. 5, the very good coincidence of experimental data and theoretical curves is observed at low temperatures 1.5–50 K, when parameters *H*_*ϕ*_ and *H*_*so*_ are properly chosen. As mentioned above, obtained parameters *H*_*ϕ*_ and *H*_*so*_ depend on dephasing times *τ*_*ϕ*_ (7) and *τ*_*so*_ (8), respectively. The calculated values *τ*_*ϕ*_ = 1 ps and *τ*_*so*_ = 0.26 ps are similar to those reported in the literature [26]. The fact has to provide an evidence for the validity of the approach. The abovementioned parameters allow obtaining the coherence length *L*_*ϕ*_ as well as the spin-orbit length *L*_*so*_ using the following relations:

10 $$ {L^2}_{\phi}= D{\tau}_{\phi}=4\frac{e}{\mathit{\hslash c}}{H}_{\phi} $$

11 $$ {L^2}_{so}= D{\tau}_{so}=4\frac{e}{\mathit{\hslash c}}{H}_{so} $$

**Fig. 6:**
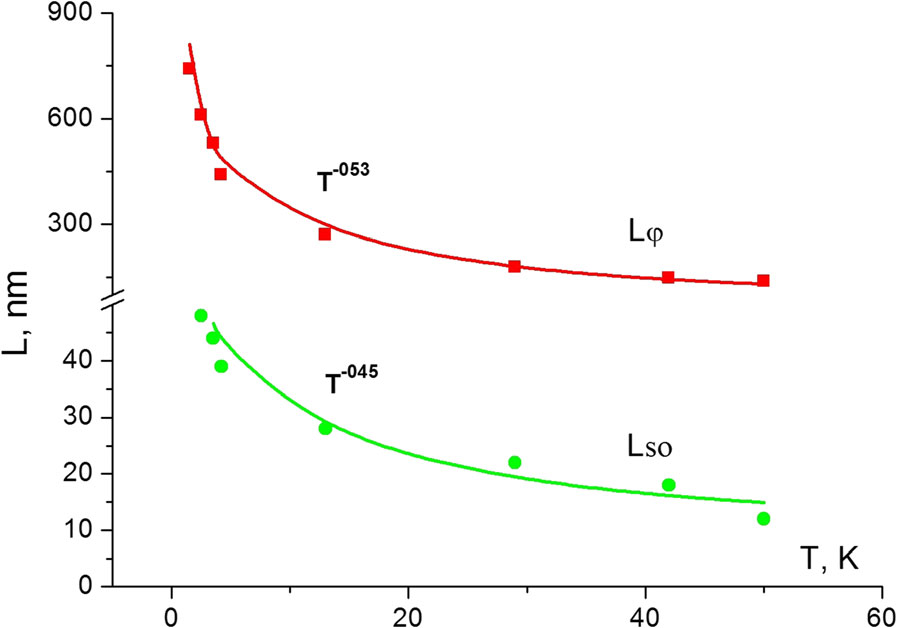
Coherence length *L*_*φ*_ and spin-orbit length *L*_*SO*_ as a function of temperature. The corresponding data points are obtained from the fitting with the two-dimensional weak antilocalization model [18] taking the electron-electron interaction and electron-phonon scattering into account. *L*_*φ*_ and *L*_*SO*_ are proportional to *T* ^− 0.53^ and *T* ^− 0.45^, respectively. It indicates the GaSb whisker conductance to occur in subsurface crystal layers

From (10–11), the temperature dependencies of *L*_*ϕ*_ and *L*_*so*_ for the studied whiskers are calculated (Fig. 6).

It is well known that the coherence length *L*_*ϕ*_ is proportional to *T* ^− 1/3^ for the one-dimensional system and *T* ^− 1/2^ for the two-dimensional one [27]. Figure 6 shows that coherence length *L*_*ϕ*_ is proportional to *T* ^− 0.53^ and the spin-orbit length *L*_*so*_ to *T* ^− 0.45^, which is very close to the *T* ^− 1/2^ dependence expected for the two-dimensional system. This fact confirms the conclusion that conductance of GaSb whiskers mainly occurs in subsurface layers of the whiskers.

Therefore, weak antilocalization and superconductivity are the main possible mechanisms responsible for the magnetoresistance observation in GaSb<Te> whiskers that is likely to originate from the whisker surface.

### Magnetoresistance Oscillations for GaSb Whiskers in High Magnetic Fields

We can analyze the whisker parameters also taking into account the SdH oscillations (Fig. 3). The magnetoresistance oscillations are periodic in 1/H. Magnetoresistance oscillation period *P* in the opposite field for quadratic dispersion law is described as follows [14]:

12 $$ P=\Delta \left(\frac{1}{H}\right)=\frac{\hslash \left| e\right|}{E_F{m}^{*} c}. $$

where *e* is the elementary charge, *ℏ* is the Planck constant, *E*_*F*_ is the Fermi energy, m* is the effective cyclotron mass, and *c* is the speed of light. In the studied GaSb whiskers doped to Te concentration near the MIT, the period was found from experimental data and reaches 0.025 T^−1^.

The analysis of SdH oscillations allows the finding of main GaSb whisker parameters. In particular, the cyclotron effective mass of electrons was determined according to the relative change in the amplitude of SdH oscillations at different temperatures *A* [14]:

13 $$ {m}_c=\frac{\left| e\right|\mathit{\hslash H}}{2{\pi}^2{k}_{\mathrm{B}}{T}_1 c} A r \cosh \frac{A\left({T}_1, H\right)}{A\left({T}_2, H\right)}, $$

where *k*_B_ is the Boltzmann constant.

This expression applies in the semiclassical region of fields assuming that it does not change with temperature and magnetic field. For weak magnetic fields, *m*_*c*_ ≈ 0.047 *m*_*o*_ is in good line with the literature data.

The mean free path of the charge carrier *l*_*e*_ could be calculated based on the equation:

14 $$ {l}_e=\frac{h{ k}_F{G}_0}{n{ e}^2}, $$

assuming a spherical Fermi surface with wave number:

15 $$ S=\pi {k_F}^2. $$

The frequency of oscillation depends on the extreme section of the Fermi surface plane *F* = *P* ^− 1^ that is perpendicular to the magnetic field for an arbitrary dispersion law and shape of the Fermi surface, according to the relation:

16 $$ F=\frac{cS}{\left| e\right| h}, $$

The frequency of oscillation for longitudinal and transverse magnetoresistance coincides, which is in good agreement with the experimental results. Substituting *F* = *P* ^− 1^ in (16), one can calculate the Fermi surface S value. Taking the calculated value *S* = 3.8 × 10^17^ m^−2^ into account, we obtained the values of wave number *k*_*F*_ = 3.46 × 10^8^ m^−1^ from (15) and mean free path of the charge carrier *l*_*e*_ = 230 nm from (14).

Thus, *l*_*e*_ *> ξ*, which confirms that GaSb is sufficiently pure to allow for odd-parity superconductors. A comparison of free path of the charge carrier *l*_*e*_ = 230 nm with phase coherence length *L*_*ϕ*_ (Fig. 6) was shown to coincide in a wide temperature range 10–60 K. On the other side, the spin-orbit length *L*_*so*_ is shown to coincide with superconductance coherence length *ξ* that indicates the spin-orbit origin of superconductivity.

According to D’yakonov-Perel spin decoherence mechanism [28],

17 $$ d{L}_{so}=\sqrt{12}{L}_{\varOmega}^2 $$

where *d* is the whisker diameter and *L*_*Ω*_ is the spin processing length with *Ω* representing the spin processing frequency under spin-orbit interaction. The spin processing length was calculated from the obtained data according to (17) and amounts to *L*_*Ω*_ = 530 nm. Taking into account the equation

18 $$ {L}_{\varOmega}=\frac{\hslash }{2{m}^{*}\alpha} $$

The Rashba spin-orbit interaction parameter *α* was calculated and it is equal to *α* = 1.66 × 10^−12^ eV m which is in good line with literature data for GaSb nanowires 3 × 10^−12^ eV m [29]. A consequence of the emergence of large Rashba field is the possibility to polarize flowing electron along the direction of this field. As a result, magnetoresistance cusps are observed in longitudinal direction, while they are not appearing in transverse one at low magnetic fields (Fig. 3).

Substituting the value of wave number *k*_*F*_ = 3.46 × 10^8^ m^−1^ and the Rashba spin-orbit interaction parameter *α* in

19 $$ {\Delta}_{so}=2{k}_F\alpha $$,

one can obtain the spin splitting energy Δ_*so*_, which was found to be Δ_*so*_ = 1.15 meV for GaSb whiskers.

Thus, we can conclude that the experimentally observed WAL in GaSb whiskers is likely to result from the 2D surface conductivity channel of the whiskers. Due to the obtained large value of spin processing length *L*_*Ω*_ = 530 nm, one could suppose that the superconducting nanoclusters are localized in subsurface layers of the GaSb whiskers.

In conclusion, one can return to the question about the nature of superconductivity in GaSb<Te> whiskers. The lower critical field *H*_c1_ shows clear linear temperature dependence from 4.2 down to 1.5 K (see inset in Fig. 2, curve 2), which could indicate a non-s-wave superconductivity but rather an unconventional superconductivity with a nodal gap structure [30]. A very low value of coherence length (*ξ*_0_ ~ 1.7 nm) like that of cuprates (*ξ*_0_ ~ 1 nm), as well as an unconventional nature of superconductivity in cuprates, could be connected with the similar features of these materials. Indeed, superconductivity of cuprates is underlined by the fact that these superconductors emerge from an antiferromagnetic Mott insulator upon carrier doping [31]. In the case of GaSb<Te> whiskers, weak antilocalization of charge carriers suggests antiferromagnetic correlation of the carriers in the metallic samples in the vicinity to MIT. The starting point is the Hubbard model in two dimensions from which we may derive an effective spin fluctuation-based interaction between electrons, which is eventually responsible for the formation of Cooper pairs. Thus, spin fluctuations could emerge due to the obtained large value of spin-orbit interaction (spin processing length *L*_*Ω*_ = 530 nm) and be responsible for competition between WAL and SC in GaSb whiskers.

## Conclusions

The magnetoresistance of n-GaSb whiskers with Te concentration 1.7 × 10^18^ cm^−3^, near the metal-insulator transition, was studied in low temperature range from 1.5–96 K and magnetic fields with induction up to 14 T. Analysis of longitudinal and transverse magnetoresistance peaks allowed to identify them as the Shubnikov-de Haas oscillations in the low temperature range 1.5–60 K. It was found that at *T* = 4.2 K, the number of transverse magnetoresistance peaks is smaller than the number of corresponding longitudinal magnetoresistance peaks.

Resistance of n-type GaSb whiskers was measured over the temperature range 1.5–300 K and magnetic field up to 10 T. The revealed sharp drop of resistance below 4.2 K indicates possible partial superconductivity in the whiskers. The magnetoconductance of these whiskers in low-field regime can be well described by a two-dimensional weak antilocalization model (WAL), where the dephasing length of the electrons follows *T* ^− 1/2^ dependence. Taking into account the WAL model as well as SdH oscillations analysis, the Rashba spin-orbit interaction parameter *α* = 1.66 × 10^−12^ eV m was calculated. A consequence of the emergence of large Rashba field is the possibility to polarize flowing electrons along the direction of this field. As a result, magnetoresistance cusps are observed in longitudinal direction, while they are not appearing in transverse one. Due to the obtained large value of spin processing length *L*_*Ω*_ = 530 nm and large whisker diameters (of about 20 μm), one can suppose that the superconductivity is created as a consequence of competition of superconductive and weak localized states in subsurface layers of the GaSb<Te> whiskers. The short coherence legnth (*ξ* (0) ~ 1.7 nm) of Cooper pairs serves as additional evidence of the competition of superconductivity and WAL in the whiskers. The origin of the superconducting state at temperature below 4.2 K is not clear, since the *T*_c_ = 1.09 K for Ga and the *T*_c_ = 3 K for possible Sb_2_Te_3_ nanoclusters, as well as the Curie temperature for GaSb<Te>, are substantially lower. Appearance of superconductivity could be explained by strong spin-orbit interaction of charge carriers in metallic phase in the vicinity to the localization threshold of MIT in the GaSb whiskers, which is known to increase substantially the Curie temperature. In order to confirm the abovementioned statement regarding the origin of the superconductive states, a thorough study of the whisker surface should be performed, which will be a task for the next investigations.

## Abbreviations

CVD: Chemical vapor deposition
LL: Landau level
MIT: Metal-insulator transition
PC: Personal computer
QDs: Quantum dots
SdH: Shubnikov-de-Haas
SQUID: Superconducting quantum interference device
WAL: Weak antilocalization
